# Supplementary material for: Content-rich biological network constructed by mining PubMed abstracts
Source: BMC Bioinformatics. 2004 Oct 8;5:147. doi: 10.1186/1471-2105-5-147 (PMC528731; doi:10.1186/1471-2105-5-147)
Supplement: Additional File 2 — The original results of the above study (non-essential files are deleted to keep the file size under the limit set by BMC bioinformatics). [file 1471-2105-5-147-S2.bz2 › chilibotAdditionalFile2/dip50/21ID9129204E79/html/TNFRSF1A_TRAF2.html]

 


 **TNFRSF1A** and **TRAF2** 
  
Found 140 abstracts in PubMed, retrieved 50.  
 

 What does Google say? 
 PDF only 
| .edu only 

---

**Interactive relationship** (e.g. stimulation, inhibition, etc)

**Neutral relationship**- These results indicate that caspase dependent cleavage of TRAF1 generates TRAF1 c fragments that are able to bind  **TRAF2** , and then sequester  **TRAF2**  from the TNF R1  [ **TNFRSF1A** ]  complex, rendering cells, at least in part, sensitive to TNF.  Ref: 11181075 Biochem Biophys Res Commun, 2001
- Apoptotic crosstalk of TNF receptors TNF R2 induces depletion of  **TRAF2**  and IAP proteins and accelerates TNF R1  [ **TNFRSF1A** ]  dependent activation of caspase 8.  Ref: 12077366 J Cell Sci, 2002
- In an in vitro binding assay, the intracellular domain of TNF R1  [ **TNFRSF1A** ]  bound TRADD, RIP and  **TRAF2**  but did not bind FADD or caspase 8.  Ref: 12721308 J Biol Chem< MedlineTA>J Biol Chem, 2002
- Importantly, we demonstrate that the cleavage product of TRAF1 coimmunoprecipitates with  **TRAF2**  that is released from the TNF R1  [ **TNFRSF1A** ]  complex in response to prolonged TNF treatment.  Ref: 11181075 Biochem Biophys Res Commun, 2001
- As  **TRAF2**  associates with most members of the TNF R  [ **TNFRSF1A** ]  family, we wished to determine how this degradation occurs.  Ref: 11909853 J Biol Chem, 2002
- TNFR  [ **TNFRSF1A** ]  associated factor 2  **TRAF2**  is an adapter protein that links several members of the TNFR  [ **TNFRSF1A** ]  family to downstream signaling pathways.  Ref: 12218092 J Immunol, 2002
- Life cell imaging and confocal microscopy revealed that both TNF R1  [ **TNFRSF1A** ]  and TNF R2 recruited the anti apoptotic factor cIAP1 in a  **TRAF2**  dependent manner.  Ref: 12077366 J Cell Sci, 2002
- Signaling via the TNF R  [ **TNFRSF1A** ]  family member CD40 has recently been shown to result in recruitment of  **TRAF2**  to plasma membrane detergent resistant microdomains lipid rafts as well as to subsequently initiate  **TRAF2**  degradation.  Ref: 11909853 J Biol Chem, 2002
- In addition, activation of TNFR1  [ **TNFRSF1A** ]  coupled to the tumor necrosis factor receptor associated factor TRAF 2  [ **TRAF2** ]  nuclear factor kappaB NF kappaB pathway alters calcium homeostasis in ASM, which appears to be a new potential mechanism underlying ASM hyper responsiveness.  Ref: 11667965 Respir Res, 2000
- Thus, TNF R2 may compete with TNF R1  [ **TNFRSF1A** ]  for the recruitment of newly synthesized  **TRAF2**  bound anti apoptotic factors, thereby promoting the formation of a caspase 8 activating TNF R1  [ **TNFRSF1A** ]  complex.  Ref: 12077366 J Cell Sci, 2002
- This interaction occurs through the leucine zipper motif common to IKKalpha, IKKbeta, and the RING finger domain of  **TRAF2** , and either IKKalpha or IKKbeta alone is sufficient for the recruitment of IKK to TNF R1  [ **TNFRSF1A** ] .  Ref: 11359906 Mol Cell Biol, 2001
- TRAF 2  [ **TRAF2** ]  is currently the best characterized TRAF family member, having a key role in mediating TNF R1  [ **TNFRSF1A** ]  induced activation of NF kappaB and JNK.  Ref: 11167129 Int J Biochem Cell Biol, 2001
- The TNF induced NF kappaB activation cascade involving TNFR1  [ **TNFRSF1A** ]  TNF receptor associated death domain TNF receptor associated factor 2  [ **TRAF2** ]   **TRAF2**  NF kappaB inducing kinase NIK IkappaBalpha kinase was interrupted at the  **TRAF2**  and NIK sites by calagualine, which would account for its suppression of NF kappaB reporter gene expression.  Ref: 12565172 Cancer Lett, 2003
- Tumor necrosis factor TNF receptor associated factor TRAF 2  [ **TRAF2** ]  is an intracellular adapter protein, which, upon TNF stimulation, is directly recruited to the intracellular region of TNF receptor 2 TNFR2 or indirectly, via TRADD, to the intracellular region of TNF receptor 1 TNFR1  [ **TNFRSF1A** ] .  Ref: 11112773 J Biol Chem, 2001

**Non-interactive relationship** (e.g. studied together, co-existance, homology, etc.)

- In this study, we further examined the interaction of the core protein with the signaling molecules of TNFR1  [ **TNFRSF1A** ] , including FADD, TRADD, and  **TRAF2** , in a human embryonic kidney cell line, HEK 293, that overexpresses the HCV core protein.  Ref: 11336543 Virology, 2001
- The initial plasma membrane bound complex complex I consists of TNFR1  [ **TNFRSF1A** ] , the adaptor TRADD, the kinase RIP1, and  **TRAF2**  and rapidly signals activation of NF kappaB.  Ref: 12887920 Cell, 2003
- TNFR  [ **TNFRSF1A** ]  associated factor 2  **TRAF2**  expression was down regulated in CD30 cells, and addition of anti TNF alpha Ab significantly reduced apoptosis in the CD30 but not the CD30 population.  Ref: 11466338 J Immunol, 2001
- Adenosine also suppressed NF kappaB dependent reporter gene expression activated by TNF or by overexpression of TNFR1  [ **TNFRSF1A** ] , TRAF 2  [ **TRAF2** ] , NIK, and p65 subunit of NF kappaB.  Ref: 12606947 Oncogene, 2003
- In response to the proinflammatory cytokine tumor necrosis factor TNF, IKK is activated after being recruited to the TNF receptor 1 TNF R1  [ **TNFRSF1A** ]  complex via TNF receptor associated factor 2  [ **TRAF2** ]   **TRAF2** .  Ref: 11359906 Mol Cell Biol, 2001
- At the same time, NF kB can be activated by TNF alpha and IL 1, which would then reach TNFR1  [ **TNFRSF1A** ] , TRADD,  **TRAF2** , MEK, JNK and c Jun activation or return to the previous IkB route.  Ref: 12542760 Tissue Antigens< MedlineTA>Tissue Antigens, 2001
- However, it is unclear how the antiapoptotic signals via  **TRAF2**  in TNF R1  [ **TNFRSF1A** ]  signaling is regulated.  Ref: 11181075 Biochem Biophys Res Commun, 2001
- Moreover, overexpression of CSN3 also inhibits NF kappaB activation triggered by proteins involved in TNF signaling, including TNF R1  [ **TNFRSF1A** ] ,  **TRAF2** , RIP, and NIK, but not by TRAF6, a protein involved in IL 1 signaling.  Ref: 11418127 FEBS Lett, 2001
- The C terminal 16 amino acid residues of TNFR2 are critical for Etk association and activation, and this Etk binding and activating motif in TNFR2 is not overlapped with the TNFR  [ **TNFRSF1A** ]  associated factor type 2  **TRAF2**  binding sequence.  Ref: 12370298 Mol Cell Biol, 2002
- When TNFalpha bound TNFR2 interacts with TNFR  [ **TNFRSF1A** ]  associated factor 2  **TRAF2** , expression of survival antiapoptotic genes is up regulated.  Ref: 12533416 Biol Reprod, 2003
- In addition, HCV core protein associates with TNF R1  [ **TNFRSF1A** ]  TRADD  **TRAF2**  signaling complex, resulting in synergistically activation of NF kappaB induced by TNF alpha.  Ref: 11374864 Biochem Biophys Res Commun, 2001
- Here we show that upon TNFalpha binding, TNFR1  [ **TNFRSF1A** ]  translocates to cholesterol and sphingolipid enriched membrane microdomains, termed lipid rafts, where it associates with the Ser Thr kinase RIP and the adaptor proteins TRADD and  **TRAF2** , forming a signaling complex.  Ref: 12753742 Immunity, 2003
- This review focuses on the molecular mechanisms that underlay signal initiation by TNF R1  [ **TNFRSF1A** ]  and TNF R2, with particular consideration of the role of TRAF 2  [ **TRAF2** ] , and highlights the importance of this molecule for the integration of such antagonizing pathways as death induction and NF kappaB mediated surviving signals.  Ref: 11167129 Int J Biochem Cell Biol, 2001
- ii NF kappaB independent protection against apoptosis by the TNF R  [ **TNFRSF1A** ]  associating factor 2  **TRAF2**  mediated recruitment of antiapoptotic proteins.  Ref: 11529675 Mol Cell Biol Res Commun, 2001
- **TRAF2**  recruitment to the TNFR1  [ **TNFRSF1A** ]  signaling complex was also disrupted by the core protein.  Ref: 11336543 Virology, 2001
- TCR independent CD30 mediated IL 13 release correlated with activation of c Jun N terminal kinase, p38 mitogen activated protein kinase MAPK, and NF kappaB, and was completely inhibited by the expression of a TNFR  [ **TNFRSF1A** ]  associated factor 2  **TRAF2**  dominant negative transgene  **TRAF2** .DN Tg, but not by that of an I kappaBalpha dominant negative transgene.  Ref: 12193714 J Immunol, 2002
- The induction of IkappaBalpha degradation by DNA damage was normal in TNF R1  [ **TNFRSF1A** ] ,  **TRAF2** , TRAF5 and FADD fibroblasts or when de novo protein synthesis was blocked.  Ref: 12654725 Genes Dev, 2003
- The constitutive expression of mRNA for TNF receptors TNFR  [ **TNFRSF1A** ] , including TNFRI and TNFRII, and the adapter molecules, such as the TNF receptor associated death domain protein TRADD, Fas associated death domain protein FADD, receptor interacting protein RIP and TNF receptor associated factor 2  [ **TRAF2** ]  TRAF 2  [ **TRAF2** ]  were analyzed by reverse transcriptase RT PCR in bone marrow samples from control, MDS and AML cases.  Ref: 12681357 Leuk Res, 2003
